# A single, clinically relevant dose of the GABA_B_ agonist baclofen impairs visuomotor learning

**DOI:** 10.1113/JP280378

**Published:** 2020-11-04

**Authors:** Ainslie Johnstone, Ioana Grigoras, Pierre Petitet, Liliana P. Capitão, Charlotte J. Stagg

**Affiliations:** 1 Wellcome Centre for Integrative Neuroimaging, FMRIB, Nuffield Department of Clinical Neurosciences, University of Oxford, Oxford, UK; 2 Department of Psychiatry, OHBA, Wellcome Centre for Integrative Neuroimaging, University of Oxford, Oxford, UK; 3 Department of Clinical and Movement Neurosciences, Institute of Neurology, University College London, London, UK; 4 MRC Brain Network Dynamics Unit, University of Oxford, Oxford, UK; 5 Department of Experimental Psychology, Wellcome Centre for Integrative Neuroimaging, University of Oxford, Oxford, UK; 6 Department of Psychiatry, University of Oxford, Oxford, UK; 7 Oxford Health NHS Foundation Trust, Oxford, UK

**Keywords:** GABA, inhibition, motor learning, neuroscience, transcranial magnetic stimulation

## Abstract

The GABA_B_ agonist baclofen is taken daily as a treatment for spasticity by millions of stroke, brain injury and multiple sclerosis patients, many of whom are also undergoing motor rehabilitation. However, decreases in GABA are suggested to be a key feature of human motor learning, which raises questions about whether drugs increasing GABAergic activity may impair motor learning and rehabilitation. In this double-blind, placebo-controlled study, we investigated whether a single 10 mg dose of the GABA_B_ agonist baclofen impaired motor sequence learning and visuomotor learning in 20 young healthy participants of both sexes. Participants trained on visuomotor and sequence learning tasks using their right hand. Transcranial magnetic stimulation (TMS) measures of corticospinal excitability, GABA_A_ (short-interval intracortical inhibition, 2.5 ms) and GABA_B_ (long-interval intracortical inhibition, 150 ms) receptor activation were recorded from left M1. Behaviourally, baclofen caused a significant reduction of visuomotor aftereffect (*F*_1,137.8_ = 6.133, *P* = 0.014) and retention (*F*_1,130.7_ = 4.138, *P* = 0.044), with no significant changes to sequence learning. There were no overall changes to TMS measured GABAergic inhibition with this low dose of baclofen. This result confirms the causal importance of GABA_B_ inhibition in mediating visuomotor learning and suggests that chronic baclofen use could negatively impact aspects of motor rehabilitation.

## Introduction

Stroke is the most common cause of adult long-term neurological disability worldwide (Feigin *et al.* 2014). Currently, one-half of all people who survive a stroke are left disabled, with one-third relying on others for activities of daily living (Stroke Association, 2016). Recovery of movement is of prime importance for stroke survivors (Pollock *et al*. 2012, 2014), with intensive physiotherapy comprsing the current ‘gold-standard’ treatment. However, many patients are left with complex problems after stroke and take a variety of medications that may interact with their potential for motor recovery (Goldstein, 1995, 1998; Goldstein *et al.* 1990). Despite early work retrospectively investigating the effect of different drug classes on motor outcomes, the effect of many individual drugs on patient’s potential for motor rehabilitation is not well understood.

It is assumed that motor recovery after stroke occurs through two main mechanisms: true recovery of function and compensation. True recovery occurs early after a stroke and reflects an interaction with spontaneous biological repair. Compensation occurs after the end of the post-ischaemic period, and the mechanisms underpinning functional improvements at this later time are considered to rely on motor learning type mechanisms (Krakauer, 2006, 2015).

Studies in healthy individuals have identified changes in motor cortex inhibition as a key physiological feature of motor learning, with decreases in concentration of the inhibitory neurotransmitter GABA being observed during motor learning (Floyer-Lea *et al*. 2006; Kolasinski *et al*. 2018) and motor plasticity (Bachtiar *et al.* 2018; Stagg *et al*. 2009; Stagg *et al*. 2011). Furthermore, interventions that decrease M1 GABA concentration [GABA] (Stagg *et al.* 2011, 2009*a*) also tend to improve performance on sequence learning tasks (Buch *et al.* 2017; Nitsche *et al*. 2003) and increase retention of visuomotor prism adaptation (O’Shea *et al.* 2017; Petitet *et al.* 2018).

In line with this, levels of GABAergic activity, as measured by positron emission tomography (Kim *et al*. 2014) and transcranial magnetic stimulation (TMS) (Bütefisch *et al.* 2003,^2008^), are reduced in stroke patients. Chronic stroke survivors also have reduced [GABA] in their ipsilesional M1 compared to age-matched controls (Blicher *et al.* 2014), and their baseline ipsilesional M1 [GABA] (Blicher *et al.* 2014) or GABA activity (Bütefisch *et al*. 2008; Kim *et al*. 2014) predicts subsequent functional improvements. Furthermore, reducing inhibition through drugs or genetic modification improved the recovery of motor function in mice post-stroke (Clarkson *et al*. 2010).

It is possible therefore that drugs increasing inhibitory signalling may impair human motor learning, and hence reduce the response to rehabilitative training in chronic stroke. One such drug is the GABA_B_ receptor agonist baclofen, which is commonly prescribed to stroke, brain injury and multiple sclerosis patients as a treatment for spasticity. This is despite advice that GABA agonists, such as benzodiazepines, should be prescribed with caution to patients with motor impairments (Hesse & Werner, 2003). Indeed, large single doses of baclofen, equivalent to the amount normally taken by patients over 2-3 days, have been shown to impair long-term potentiation-like plasticity in M1 of healthy volunteers (McDonnell *et al*. 2007), as well as impair performance on a torque-tracking task (Willerslev-Olsen *et al.* 2011).

In the present study, we performed a within-subjects, double-blind, placebo-controlled cross-over study of the effect of 10 mg of baclofen, equivalent to the normal dose taken by patients 2-3 times per day, on visuomotor and sequence learning in young healthy participants. Visuomotor learning and retention were evaluated using a joystick-based visuomotor learning (VML) task; and sequence learning was assessed by a serial reaction time task (SRTT). In addition, TMS measures of motor cortex excitability, as well as activity at GABA_A_ and GABA_B_ receptors, were recorded at multiple timepoints as an index of the efficacy of baclofen on receptor activation.

Visuomotor learning measures improvements in the accuracy of sensorimotor mapping, such as how movement space translates into visual space, in response to a perturbation, whereas motor sequence learning focuses on assessing participant’s ability to link together a sequence of well-practiced movements. Improvements in performance on both of these tasks can be considered as model processes for the types of learning that may be important in motor rehabilitation following brain injury. We predicted that baclofen would significantly impair performance on both motor tasks by altering the underlying processes controlling the behaviour, and also that these changes would correlate with baclofen-induced changes in long-interval intracortical inhibition measures (150 ms) of GABA_B_ activity.

## Methods

### Ethical approval

This research was approved by the Central University Research Ethics Committee (CUREC), Oxford University (Ref: R55534/RE001), in line with the *Declaration of Helsinki,* 2013, except for registration on a database. All participants provided their written informed consent to all experimental procedures.

### Participants

Twenty healthy volunteers (nine male, aged 21-32 years, mean age 24.9 years) participated in this study. All participants were right-handed as assessed by the Edinburgh Handedness Inventory (Oldfield, 1971) and did not play a musical instrument to a high level, defined as reaching ABRSM grade 5 (or equivalent) and still in regular practice. Participants reported no history of neurological diseases, head injuries or other contraindication for TMS. Participants were not taking any prescription medicines at the time of testing, with the exception of the oral contraceptive pill.

### Experimental overview

**Figure 1:**
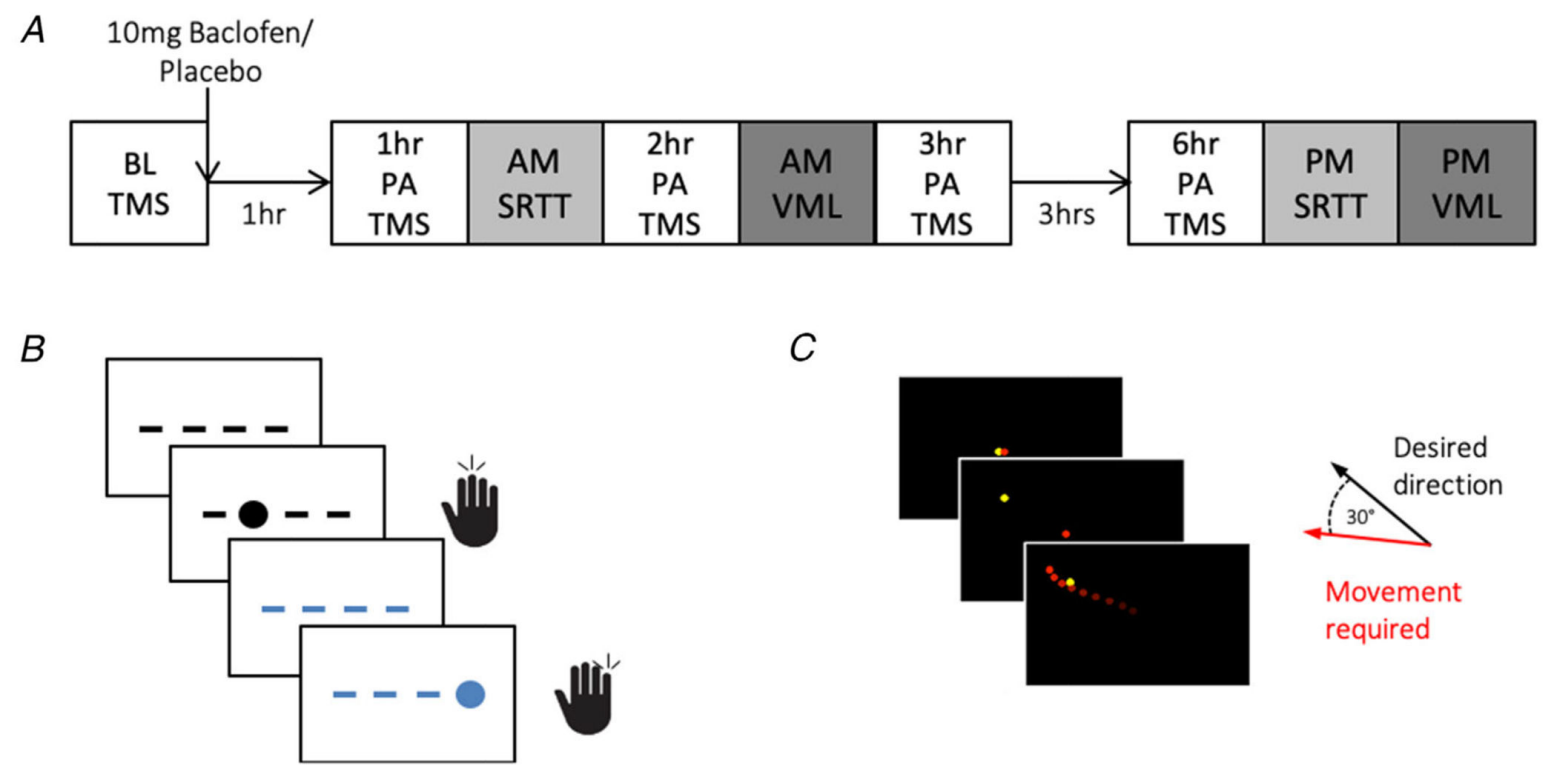
Study timeline and motor tasks *A,* timeline of testing day showing the transcranial magnetic stimulation (TMS) measures taken at baseline (BL) and several timepoints post-administration (PA) of drug. *B,* serial reaction time task(SRTT) screen, showing random cues in black and sequence cues in blue. *C,* visuomotor learning task (VML) screen during feedback on trial. Participant must add 30° anti-clockwise compensation to movements for the cursor to hit the target.

Participants attended two identical testing days, 1 week apart, in which a single dose of baclofen (10 mg) or placebo (lactose/sucrose), over-encapsulated in a gelatine capsule, was given as an intervention. Session order was counterbalanced across the group. An outline of the experimental sessions is provided in Fig. 1.

All testing days began with safety screening at 09.00 h, followed by baseline mood questionnaire measures, base-line TMS measures and task training. Participants took a capsule orally at ~ 10.00 h, followed by a 1 h waiting period to allow the baclofen to reach peak plasma concentration (*T*_max_ = 1-3 h) (Hulme *et al.* 1985). Participants then completed motor tasks, mood questionnaires and TMS measures over the next 2 h. This session, referred to as the AM session, finished by 13.00 h. Participants remained in the testing centre for the next 3 h, during which time they sat quietly with one of the experimenters. Six hours post-administration (~ 16.00 h) participants performed another set of motor task assessments, mood questionnaires and TMS measures. This PM session was designed to assess retention of motor skills once the drug had decreased below effective levels because the half-life of baclofen in plasma is around 3-4 h.

### Motor tasks

All tasks were presented using a laptop monitor (Latitude E5540; Dell, Round Rock, TX, USA) at a distance of ~40 cm. The laptop was placed on a 15 cm high wooden shield box, designed to occlude the participant’s hand from their view. The VML task was presented using PsychoPy v2 (Peirce, 2007) and the SRTT was written with Psychtoolbox (PTB-3) (Brainard, 1997) for MATLAB, and displayed using MATLAB, R2014b (The Mathworks Inc., Natick, MA, USA) (SRTT).

#### VML

This task measured participant’s ability to learn a perturbation of visuomotor mapping. Full versions of the task were run twice during each testing day: once at ~2.5 h post-administration (PA) in the AM session; and once at 6.5 h PA in the PM session. Participants also practiced on a short (<5 min) version of the VML task, without perturbation, before drug administration.

Participants used a joystick (Attack 3; Logitech, Lausanne, Switzerland) in their right hand to control a red cursor presented onscreen (60 Hz sampling rate). At the beginning of each trial, with the joystick in the neutral position, the cursor was positioned in the centre of the screen along with a yellow target dot. After a delay of 1 s, the yellow target disappeared from the central location and reappeared at one of eight equally spaced locations on an imaginary circle with a radius of 4.5 cm around the centre of the screen (N, NE, E, SE, S, SW, W, NW). The targets appeared in each location randomly within each set of eight consecutive trials. The participant was instructed to make rapid ‘shooting’ movements to guide their cursor through the target, similar to previously described tasks (Galea *et al.* 2011). After 2 s, the target returned to the central position (Fig. 1B).

During the task there were feedback on (FB-on) and feedback off (FB-off) blocks. For trials during the FB-on blocks, the participant could see their cursor until it moved off screen, meaning they could see the trajectory of their movements and proximity to the target. In FB-off trials, the cursor disappeared as soon as the participant moved the joystick, meaning they could not see the trajectory of their movement. Each block consisted of 40 trials (five repeats of the eight different trial target locations). Both AM and PM task versions consisted of 5 FB-on blocks alternating with 5 FB-off blocks. During the AM session, a 30° clockwise deviation was induced during the FB-on trials. Participants therefore began with a 30° error in their movements, which they could reduce through learning a transformation in visuomotor mapping. Recording the error in the FB-off trials in the AM session, where visual feedback and perturbation were removed, gave a measure of the visuomotor after-effect. Error in the PM session, where no perturbation was present, measured the degree to which participants unlearnt the mapping and retained the visuomotor after-effect. Previous studies have shown that higher GABA concentrations correlate with decreased visuomotor after-effect and decreased retention of the learnt mapping when tested on the same task later (O’Shea *et al*. 2017; Petitet *et al*. 2018).

#### VML data analysis

Performance on this task was quantified by recording the angular error (°) between the target and the point at which the cursor crossed through the imaginary circle on which the targets were placed. Individual trials were removed from further analysis if the error was greater than ±67.5°, indicating a movement towards the wrong target.

Poor quality data necessitated the removal of data from a number of participant’s sessions from the analysis. Problems with the joystick to PC connection resulted in a failure to record movements in one dimension during some task runs. Additionally, one session of data was removed for two subjects because they had rotated the joystick. In total, two participants had both AM sessions excluded from further analysis, and another three had one session removed. PM sessions were excluded for those participants whose AM sessions had been removed. One further participant had one PM session removed as a result of data recording failure.

#### State-space modelling of the AM VML data

Although the average error across trials provides a way to examine the behaviour on the VML task, it is also possible to apply a model-based approach to estimate the underlying processes driving the observable behaviour. Here, we applied a state-space model with one parameter controlling the rate of learning and another controlling rate of forgetting (Donchin *et al*. 2002, 2003; Smith *et al*. 2006). Parameters were estimated by minimizing the mean square error with *fmincon* in MATLAB, R2018a. Only subjects who had complete data sets from both AM sessions were included in this analysis (*n* = 14).

e(n) = d(n) - x(n)

x(n) = A.x(n - 1) - V (n) .B.*e* (n - 1)

0 < A < 1; 0 < B < 1

Where *e*(*n*) is the angular error on trial *n*, *d(n)* is the perturbation (rotation); *x(n)* is the degree of visuomotor learning; *A* is the retention rate; *B* is the learning rate; and *V(n)* reports whether feedback is on (*V* = 1) or off (*V* = 0). The model fit to individual’s data with a mean *r^2^* = 0.359.

#### SRTT

To assess motor sequence learning, participants performed a visually cued reaction time task with their right hand based on that used by Nissen & Bullemer (1987). The task was performed twice in each testing day, once at ~1.5 h PA in the AM session and once at 6 h PA in the PM session.

During the task, circular cues appeared sequentially at one of four locations arranged horizontally on the screen. Participants were instructed to respond to these cues as quickly and accurately as possible by pressing the corresponding button on an external 4-button box (OTR-1 × 4-CR; Current Designs Inc., Philadelphia, PA, USA) using the four fingers of their right hand (Fig. 1C).

The task was separated into random blocks, made up of 48 button presses with cues presented in black against the white screen, and sequence blocks, consisting of five repeats of a 12-item sequence with cues presented in blue. Participants were informed of the presence of the sequence, but not its length or structure, prior to beginning the task. In the AM session. the task was split into three sections (i.e. familiarization, training and post-training) with 1 min breaks in between. The familiarization section contained one sequence block between two random blocks, the training section contained seven sequence blocks and the post-training section contained one sequence block flanked by random blocks. The PM session SRTT had three sequence blocks bookended by two random blocks. The sequences differed between the first and second testing day (sequence day 1: 312 423 214 134, sequence day 2: 342 131 241 432). For one participant, the computer crashed during the familiarization section on day 1 and so this participant performed the task using a third sequence (sequence 3: 231 434 124 213).

#### SRTT analysis

For the SRTT, participant’s change in skill across time was the primary outcome of interest. Learning the SRTT causes reductions in magnetic resonance spectroscopy (MRS)-measured GABA concentration (Kolasinski *et al*. 2018), we therefore predicted that, by agonizing GABA_B_ receptors with baclofen, we might see reductions in skill change.

Response time was calculated as the time from cue onset to button press. In line with previous studies using the SRTT (Robertson *et al*. 2004), incorrect responses and RTs outside of 2.7 SDs of the mean value for the block were excluded. Initial AM skill was calculated as the difference in mean RT between the random and sequence blocks during familiarization. The same approach was used to calculate the final AM skill using the data from the post-training section. Skill at the beginning and end of the PM session (initial PM skill, final PM skill) was calculated by subtracting the first sequence block from the first random block, and the final sequence from the final random block in the PM session.

### TMS

All TMS data were acquired using a monophasic DuoMAG machine connected to a 70 mm figure-of-eight coil (Rogue Resolutions, Cardiff, UK). The left M1 first dorsal interosseous (FDI) motor hotspot was targeted, comprising the position where motor-evoked potentials (MEPs) could be elicited in the right FDI muscle at the lowest stimulator intensity. The TMS coil was held at 45° to the midsagittal line with the handle pointing posteriorly. The hotspot was marked by drawing guidelines onto the participants scalp to allow for the same coil placement across the day’s measurements.

Surface EMG was recorded using disposable neonatal ECG electrodes (Henley’s Medical, Welwyn Garden City, UK) from the FDI of the right hand using a belly-tendon montage with a ground electrode over the ulnar styloid process. Signals were sampled at 5 kHz, amplified, filtered (10 Hz to 1 kHz), and recorded using a Digitimer D440 4-Channel Isolated Amplifier (Digitimer, Welwyn Garden City, UK), a CED micro1401 A/D converter (Cambridge Electronic Design, Cambridge, UK) and Signal software, version 3.13 (Cambridge Electronic Design). A Digitimer Humbug Noise Eliminator was used to reduce 50 Hz noise in the EMG recordings (Digitimer).

Corticospinal excitability (CE), short-interval intra- cortical inhibition (SICI_2.5ms_), and long-interval intra- cortical inhibition (LICI_150 ms_) measures were collected at five timepoints per testing day: before drug administration (Baseline), 1h PA, 2h PA, 3h PA and 6h PA CE was assessed by quantifying the amplitude of MEPs produced in response to stimulation of the intensity required to elicit MEPs of ~ 1 mV peak-to-peak amplitude in the resting FDI muscle at beginning of the testing session, henceforth 1 mV-MT. At each time point, 10 single-pulse MEPs were recorded at the initial 1 mV-MT _init_ immediately before each TMS run. This intensity was not varied across the session.

Two paired-pulse protocols were performed in the study. SICI with an interstimulus interval (ISI) of 2.5 ms was recorded to assess GABA_A_ receptor activity (Di Lazzaro *et al.* 2006; Kujirai *et al.* 1993; Stagg *et al.* 2011a), where the conditioning stimulus was set at 70% of the active motor threshold (aMT), comprising the intensity needed to evoke a 200 μV peak-to-peak MEP in five of 10 trials when subjects maintained ~30% of the maximum contraction of the FDI (aMT). The test stimulus was set at 1 mV-MT. LICI with an ISI of 150 ms was also recorded as a measure of GABA_B_ inhibition (McDonnell *et al*. 2006, 2007; Müller-Dahlhaus *et al*. 2008) and both the test and conditioning stimuli were set at 1 mV-MT. The 1 mV-MT used in these measurements was adjusted (1 mV-MT_adj_) at each measurement timepoint to account for potential changes in motor cortex excitability as a result of drug administration or motor performance. At each timepoint, the SICI and LICI protocols consisted of 12 1 mV-MT_a_dj and either twelve SICI_2.5_ ms paired-pulses or twelve LICI_150 ms_ paired-pulses delivered in a pseudo-randomized order.

#### TMS analysis

TMS data were collected from all but two participants, one because of equipment failure, and another as a result of high TMS thresholds. LICI_150 ms_ measurements were not recorded from a further two participants because of technical issues.

Data cleaning was performed in line with previous TMS studies conducted in the group (Nowak *et al.* 2017). Trials were excluded if there was precontraction in the right FDI muscle (EMG amplitude >0.15 mV in the 30 ms preceding the pulse). Peak-to-peak amplitude for each MEP was quantified and MEPs greater than 3.5 mV or outside of 2 SDs for mean for each measurement type, for each timepoint, for each participant, were rejected. These MEPs were then run through a single iteration Grubbs’ test with a significance level of 0.05 to exclude any remaining significant outliers. SICI_2.5 ms_ and LICI_150 ms_ are expressed as a fraction of the mean paired-pulse MEP amplitude over the mean single-pulse MEP amplitude (1 mV-MT_adj_). TMS data was analysed using repeated measures ANOVAs and *t* tests because it was assumed that carry-over effects between testing days were improbable and, for participants in whom TMS measures were recorded, there were no missed data points. *P* < 0.05 was considered statistically significant. Marginal differences with *P* < 0.10 are also reported.

### Other assessments

#### Bond–Lader visual analogue scale (VAS)

The participants were asked to complete a paper Bond-Lader VAS three times on each day: before treatment administration (Baseline), 1 h PA and 6 h PA. Participants gave a subjective account of various aspects of their mood by marking their current state of mind on 16 different 100 mm analogue scales between two opposing emotions (e.g. Alert-Drowsy, Happy-Sad).

For each VAS question, the mean of the two baseline measurements from each day for each participant was calculated. To reduce the dimensionality of the data, principal component analysis was used on these 16 baseline measures to define two principle components that explained the most variance in a participant’s mood. The first component was predominantly influenced by the participant’s score for feeling alert, strong, clear-headed, well co-ordinated, energetic, quick-witted and attentive, whereas the second component was predominantly influenced by the participant’s score for feeling strong, tranquil, relaxed and happy. The first component will henceforth be referred to as the ‘alertness component’, and the second component as the ‘hedonic component’ The weights for each question, for each component, were applied to the data to calculate the scores for the two components, for each participant, at each timepoint, on each testing day.

#### Rey auditory-verbal learning test (AVLT)

Basic memory tests were performed to assess for general effects of baclofen on memory. Participants were asked to complete the AVLT ~2 h after drug administration. The experimenter read the participant a list of 12 words (List A) at a rate of 1 Hz (Rey, 1958; Spreen *et al.* 1998). The participant was then asked to recall as many words as they could remember. This procedure was carried out five times using the same list. Participants were then presented with a second list of words (List B) that they were given one chance to recall. Following this, participants were asked to recall List A for a sixth time (immediate list recall). After a further 10 min, participant’s recall of List A was assessed again (delayed list recall).

Performance on this task was quantified as the number of words participants could recall on the first five repetitions of List A, out a total of 60, comprising AVLT immediate recall. The ratio of the number of words correctly recalled in the delayed recall probe with respect to the number recalled at final immediate repeat was also caclulated, comprising AVLT delayed/immediate. One participant was excluded from analysis because they did not understand the task instructions on the first day. Different versions of List A and List B were used on each testing day.

#### Digit span test

Participants performed the digit span test, both forwards and backwards, ~2 h after drug administration. During the forward section, the participants were instructed to repeat the digit sequence in the same order that they heard it from the researcher, whereas, during the backward section, they had to repeat the digit sequence in the reverse order.

Both forward and backward sections began with sequences of two digits in length. Participants were assessed on two sequences of each length and, if the participant recalled at least one of the sequences correctly, then the task would proceed and they would be read sequences with an additional digit. Performance at this task, for both the forward and backward sections, was measured by recording the highest number of digits at which the participant correctly recalled at least one sequence.

#### Blinding assessment

At the end of both testing days, the participant and the experimenter who carried out the TMS measurements completed a blinding assessment form. On this form, the participant/experimenter reported their guess of the treatment (baclofen/placebo) and marked their certainty about this guess on a 100 mm analogue scale.

Blinding indices (BI) were calculated using the method outlined in Bang *et al.* (2004). This calculated an index on a scale of -1 to 1 for each drug condition, where -1 indicates completely opposite guessing, 1 indicates completely perfect guessing and scores between ±0.2 indicate adequate blinding.

Only the data from day 2 were included in this analysis because this was considered to be the time when participants would have the most information to make a guess about their drug condition. If the participant or the experimenter indicated that they were less than 50% certain about a response, this was coded as a ‘don’t know’ response for the blinding index calculation.

### Statistical analysis

All pre-processing was performed using MATLAB, R2018a (The Mathworks Inc.) and statistical tests were performed using SPSS, version 25 (IBM Corp., Armonk, NY, USA). Outcome measures from the motor tasks were analysed using linear mixed models (LMM) to enable drug effects to be examined at the same time as controlling for other potential confounding variables, such as day.

To create measures of the effect of baclofen on specific tasks and neurophysiological assessments for use in correlational analyses, we performed placebo correction by subtracting the measures taken in the placebo session from the same measures in the baclofen session. From the SRTT task, the placebo-corrected change in skill across the AM session was calculated. From the VML task, the placebo-corrected mean angular error from the FB-on and FB-off blocks of the AM session, as well as the mean change in angular error for the two different FB block types (block 5 error- block 1 error), was calculated, as were the placebo-corrected parameters from the state-space model. Finally, placebo-corrected TMS measures were calculated, by taking the average of the TMS measures during drug peak plasma concentration (1 h PA, 2 h PA and 3 h PA) and subtracting the baseline measure.

## Results

### Baclofen significantly reduced AM visuomotor aftereffect and increased PM washout of learnt visuomotor mapping

First, we wished to investigate the effects of GABA_B_ agonism on angular error measurements in the VML task. Analysis was performed separately for the FB-on and FB-off blocks of the AM and PM sessions. Average angular error measurements for each of the 5 FB-on and FB-off blocks were calculated and input into LMMs [Fixed effects of Time (B1, B2, B3, B4, B5), Drug (baclofen, placebo), Day (1, 2), Time by Drug interaction, Day by Time interaction; random effect of Participant].

**Figure 2:**
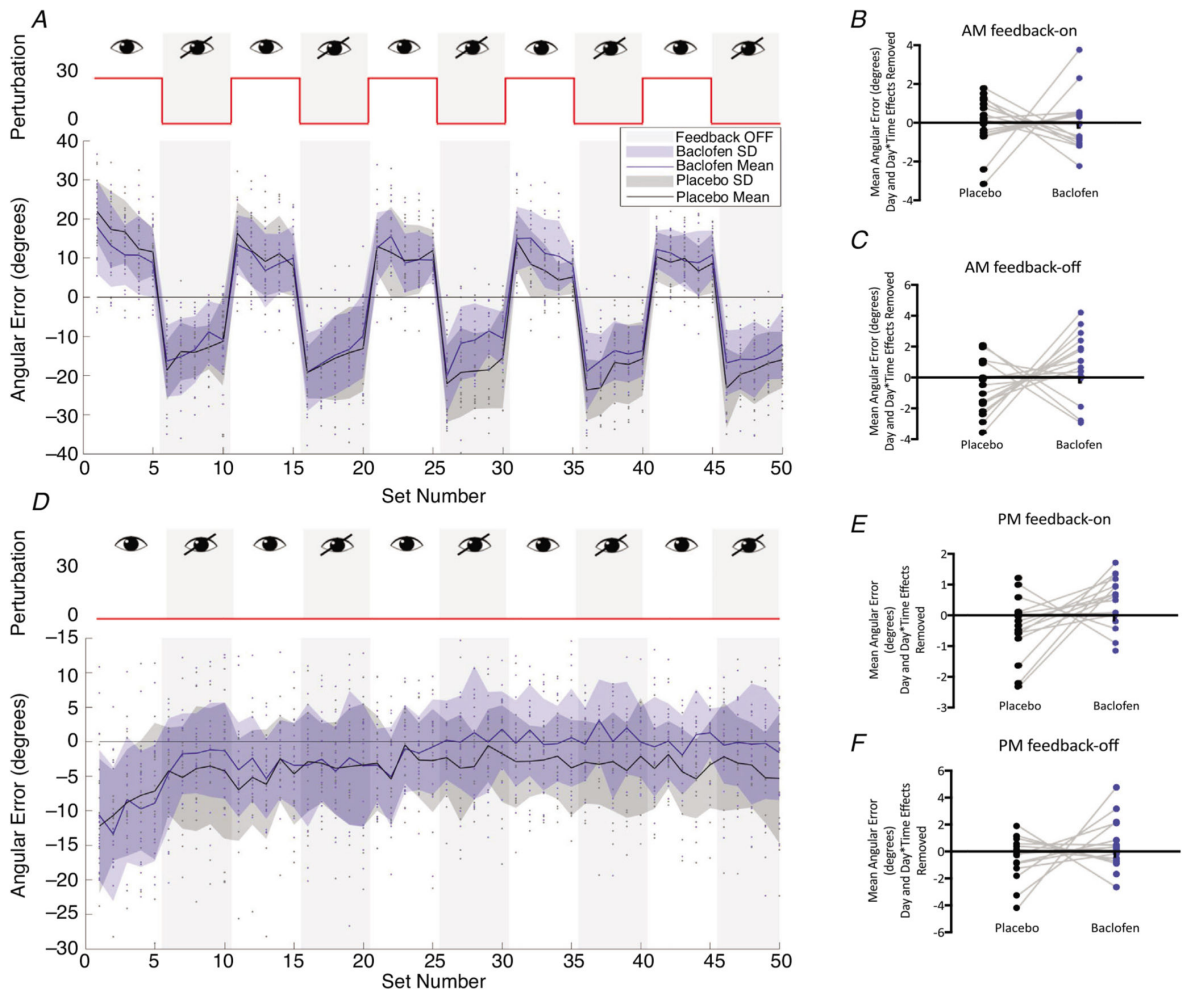
Individual subject and mean (**+**SD) group data from the VML task *A,* group AM session data from visuomotor learning task. Showing a significant reduction in overall angular error in the baclofen condition across the FB-off blocks (linear mixed model, *n* = 18). Dots show individual subject data points, a line signifies mean angular error and a shaded area signifies standard deviation. *B,* mean angular error in the AM FB-on condition for each participant. *C,* mean angular error in the AM FB-off condition. *D,* group PM session data, again demonstrating an overall reduction in angular error in the FB-on blocks with baclofen (linear mixed model, *n* = 18). *E,* mean angular error in the PM FB-on condition for each participant. *F,* mean angular error in the PM FB-off condition. All individual participant data (i.e. in *B*, *C*, *E* and *F*) are controlled for the effect of Day and Day × Time. Individual data charts displayed for data visualization purposes only, not for statistical inference.

In the AM session FB-on blocks, participants were able to learn and decrease their error across the blocks of the task (Time: *F*_4_,_138_._0_ = 6.125, *P* < 0.001); however, there was no effect of baclofen on overall angular error (Drug: *F*_1,138.0_ = 0.006, *P* = 0.940) or the change in the error over the blocks (Drug by Time interaction: *F*_4,138.0_ = 1.062, *P* = 0.378). There was also no effect of testing Day (*F*1,15.3 = 0.744, *P* = 0.402), nor a significant Dayby Time interaction (*F*_4,138.0_ = 0.577, *P* = 0.680) (Fig. 2A and B).

We then examined the effects of baclofen on visuomotor aftereffect (i.e. the degree to which participants continued to display the learnt transformation in visuomotor mapping when feedback and perturbation was removed) using a separate LMM. As would be expected, in the FB-off blocks of the AM session, participants showed a significant increase in visuomotor aftereffect (i.e. increasing error) across the blocks of the task (Time: *F*_4,138.0_ = 3.423, *P* = 0.011). In the baclofen condition, the overall error across the FB-off blocks was significantly less negative, indicating a reduction in visuomotor aftereffect (Drug: *F*_1,137.8_ = 6.133, *P* = 0.014) (Fig. 2A and C). There was no difference in how this error changed across blocks between the drug conditions (Drug by Time interaction: *F*_4,138.0_ = 0.366, *P* = 0.833) or in any other of the effects modelled (Day: *F*_1,16.0_ = 0.059, *P* = 0.812; Day by Time interaction: *F*_4,138.0_ = 0.302, *P* = 0.876).

As expected, in the PM session, we observed a significant loss of learnt transformation in visuomotor mapping, measured as a decrease in angular error, across the FB-on blocks (Time: *F*_4,123.8_ = 37.383, *P* < 0.001). There was an overall increase in the washout of the learnt visuomotor mapping (i.e. less angular error, or less retention of the learnt mapping) in the baclofen condition (Drug: *F*_1,130.7_ = 4.138, *P* = 0.044) (Fig. 2D and E). There were no other significant effects in the model (Day: *F_1,13.6_*= 4.138, *P* = 0.356; Drug by Time interaction: *F*_4,123.8_ = 0.796, *P* = 0.530; Day by Time interaction: *F*_4,123.8_ = 0.860, *P* = 0.490).

Finally, to examine retention of the visuomotor after-effect, the FB-off blocks in the PM session were analysed (Fig. 2D and F). There were no significant effects (Main effect of Time: *F*_4,124.0_ = 2.418, *P* = 0.052; Main effect of Drug: *F*_1,130.8_ = 12.891, *P* = 0.091; Main effect of Day: *F*_1,13.9_ = 0.740, *P* = 0.564. Drug by Time interaction: *F*_4,124.0_ = 0.322, *P* = 0.863; Day by Time interaction: *F*4,124.0 = 1.392, *P* = 0.24).

To further characterize the change in visuomotor learning dynamics induced by baclofen, a state-space model was fit to the AM VML data. LMM analysis of the learning and retention parameter values [Fixed effects of Drug (baclofen, placebo), Day (1, 2), random effect of Participant] revealed that there was a trend towards a significant effect of Drug on the retention parameter (A) (Drug: *F*_1,13_ = 3.570, *P*= 0.081) driven by an decrease in the retention parameter in the baclofen condition. There was also a significant effect of Day (*F*_1,13_ = 6.631, *P* = 0.023). The same analysis revealed no significant effect of Drug (*F*_1,13_ = 0.200, *P* = 0.662), nor Day (*F*_1,13_ = 3.810, *P* = 0.071) on the learning rate parameter (B).

### Baclofen did not significantly impact change in skill measures on the SRTT

We then examined whether this behavioural effect of baclofen would also be seen in the SRTT. Accordingly, we first needed to assess whether baclofen had any effect on simple reaction times. We therefore input the mean RT from all random blocks in the AM session into an LMM [Fixed effects of Drug (baclofen, placebo), Day (1, 2); random effect of Participant]. Baclofen did not cause any differences in RT to random cues (Drug: *F_1,18_* = 1.060, *P* = 0.317), although there was a significant effect of day, where participants made faster responses on the second testing day (Day: *F*_1,55_ = 36.51, *P* < 0.001). We therefore included Day as a factor in all subsequent LMMs.

**Figure 3:**
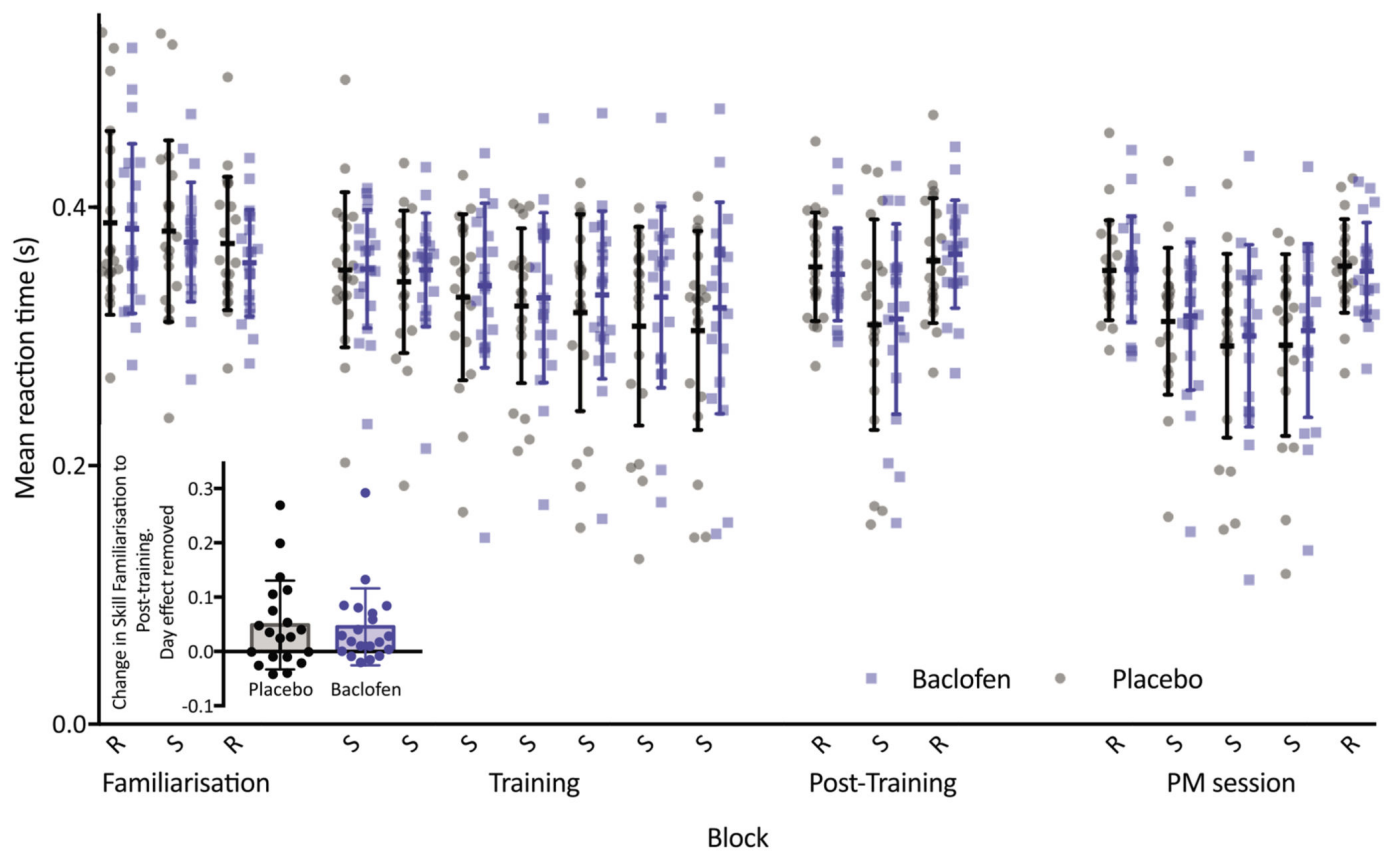
Individual subject and mean (**+**SD) group data from the SRTT task Individual subject data points and mean reaction time (errors show SD) for each block of the SRTT There was learning on the task in both the drug conditions, but no significant difference in the change in skill (RT on random blocks, –RT on sequence block) across the AM session (inset) (linear mixed model, *n* = 20), across the PM sessions, or in skill retention between the AM and PM sessions. Bar chart displays data controlled for the effect of Day; the bar chart is provided for data visualization purposes, not for statistical inference.

To examine the effect of drug on change in SRTT learning, initial AM skill and final AM skill were input into an LMM [fixed effects of Time (initial AM, final AM), Drug (baclofen, placebo), Day (1,2), Time by Drug interaction, Day by Time interaction; random effect of Participant]. As would be expected, skill on the task improved with training (Time: *F*_1,55_ = 19.63, *P* < 0.001). However, baclofen had no effect on the rate of learning in the AM session (Drug by Time interaction: *F*_1,55_ = 0.026, *P* = 0.872). There was a significant effect of Day on the change in skill, driven by a greater improvement in skill on the second day (Day by Time interaction: *F*_1,55_ = 5.498, *P* = 0.023). There were no other significant effects (Drug: *F*_1,55_ = 0.082, *P* = 0.776. Day: *F*1,55 = 0.012, *P* = 0.912) (Fig. 3).

Baclofen did not impact skill retention between the AM and PM [LMM: Fixed effects of Time (final AM, initial PM), Drug (baclofen, placebo), Day (1, 2), Time by Drug interaction, Day by Time interaction; random effect of Participant] (Drug by Time interaction: *F*_1,55_ = 0.006, *P* = 0.936). Similarly, baclofen had no impact on later relearning of the task in the PM session [LMM: Fixed effects of Time (initial PM, final PM), Drug (baclofen, placebo), Day (1, 2), Time by Drug interaction, Day by Time interaction; random effect of Participant] (Drug by Time interaction: *F*1,55 = 0.411, *P* = 0.524).

### Correlations between drug-induced behaviour changes

Finally, we aimed to explore whether baclofen-induced behavioural effects generalized across the two motor learning tasks, even in the absence of a significant behavioural effect in the SRTT. We reasoned that a lack of significant effect on the SRTT might be the result of either: no modulation by baclofen, in which case the drug-induced change in RTs would not correlate with the drug-induced change in VML metrics, or a difference in signal to noise in our two tasks, in which case the drug-induced behavioural changes might correlate.

#### Significant correlations between effects of baclofen on the motor tasks

We found significant Pearson’s correlations between the baclofen-induced change in the SRTT learning and the VML measures that were significantly influenced by baclofen. There was a significant correlation between the non-significant baclofen effect on SRTT learning and the significant baclofen effect on VML aftereffect (*r*_14_ = -0.674, *P* = 0.006) and there was a trend towards correlation with the retention parameter from the state space model (*r*_14_ = -0.505, *P* = 0.055). The correlations between baclofen effect on SRTT learning and the VML measures not influenced by baclofen, such that visuomotor learning on the VML (*r*_14_ = 0.280, *P* = 0.313) and learning parameter (*r*_14_ = -0.221, *P* = 0.429) were not significant. Significant correlations met the Bonferroni corrected significance level (α = 0.013).

### Effects of baclofen on TMS-derived neurophysiological metrics

We then wanted to investigate the neurophysiological effects of baclofen. First, we confirmed that there were no systematic differences between the sessions before drug administration in terms of spTMS amplitude (*t*_17_ = 0.474, *P* = 0.642). There were also no significant baseline differences in SICI_2.5 ms_ measures of synaptic GABA_B_ mediated inhibition (*t_17_* = 0.202, *P* = 0.842) or LICI_150 ms_ measures of GABA_B_ mediated inhibition (*t*_14_ = 0.700, *P* = 0.495).

The combined single-pulse 1 mV-MT_adj_ MEP measure was stable across the testing days (repeated measures ANOVA, two levels of Drug: placebo, baclofen; five levels of time: Baseline, 1 h PA, 2 h PA, 3 h PA and 6 h PA), although there was a trend towards a main effect of Time (*F*_4,68_ = 2.206, *P* = 0.078) and a trend towards a significant Time by Drug interaction (*F*_4,68_ = 2.501, *P* = 0.050). No main effect of Drug (*F*_1,17_ = 1.004, *P* = 0.330) was observed. Finally, we ensured that our paired pulse measures resulted in the expected inhibitory effect of SICI_2.5 ms_ (*t*_35_ = 5.19, *P* < 0.001) and LICI_150 ms_ (*t_29_* = 3.776, *P* < 0.001).

#### Baclofen significantly reduced overall corticospinal excitability

Having established that the TMS measures were interpretable, we then investigated the effects of baclofen on the TMS measures. Given that there was no difference in MEP amplitude between the drug and placebo conditions at baseline, the CE measures for the subsequent timepoints (1 h PA, 2 h PA, 3 h PA and 6 h PA) were then normalized to baseline for further analysis.

**Figure 4:**
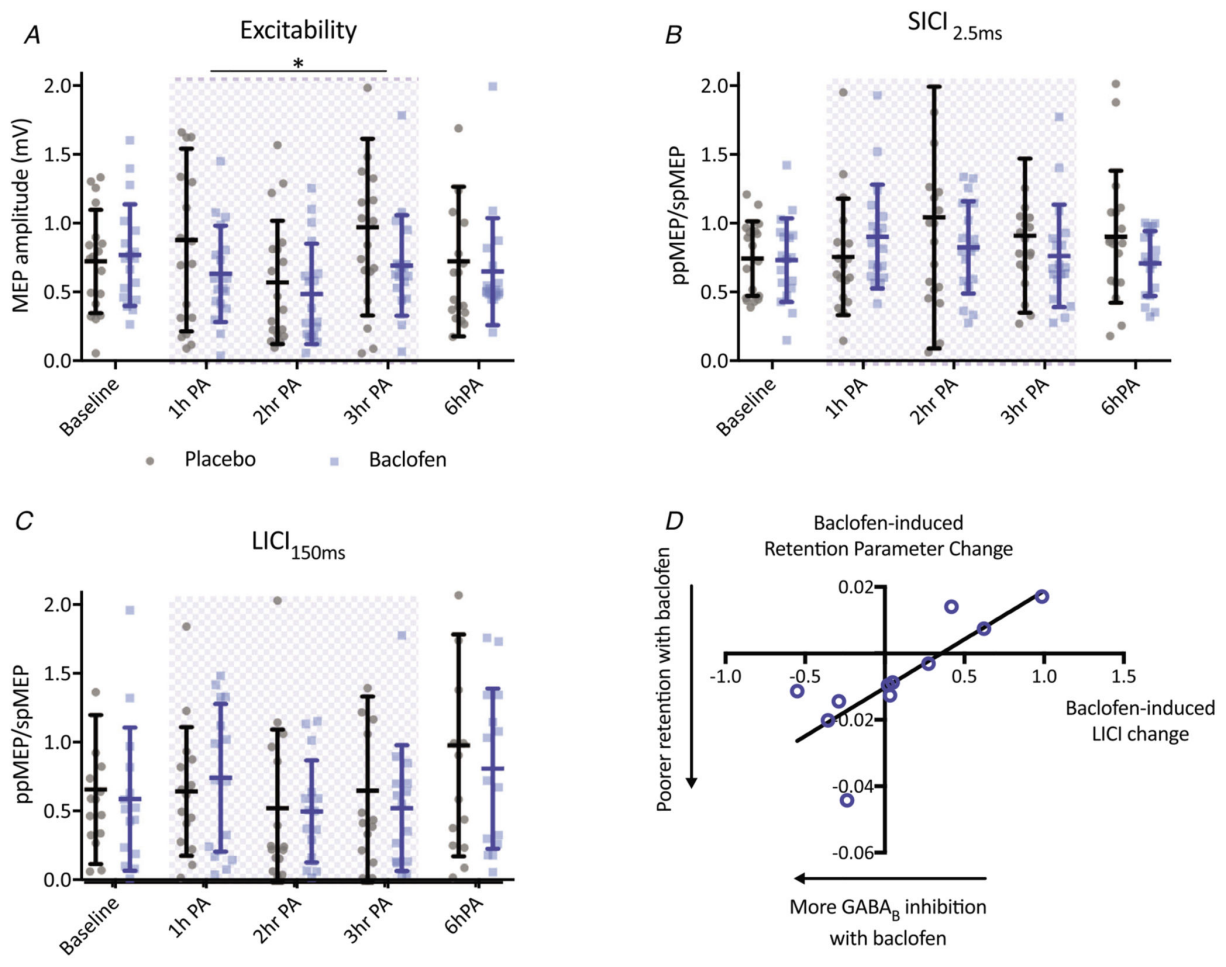
Individual subject and mean (**+**SD) group TMS data, and TMS-behaviour correlation *A,* decrease in corticospinal excitability during baclofen peak plasma concentration (blue hatch) (repeated measures ANOVA, *n* = 18). Excitability returned to normal levels after drug washout at 6 h PA (paired samples *t* test, *n* = 18). Individual subject data points are shown, along with the mean ± SD of MEP amplitude at each time point. *B,* no change in GABA_A_ receptor inhibition with baclofen (repeated measures ANOVA, *n* = 18). *C,* no change in TMS-measured GABA_B_ receptor inhibition with baclofen (repeated measures ANOVA, *n* = 15). *D,* significant Pearson correlation (*r* = 0.782, *n* = 11, *P* = 0.004) between baclofen-induced increases in GABA_B_ inhibition and the baclofen-induced retention parameter from the state space model.

The normalized measures from the three timepoints during the AM session were analysed using a repeated measures ANOVA with a within-subjects factor of Drug (baclofen, placebo) and a within-subjects factor of Time (1 h PA, 2 h PA and 3 h PA). This revealed a significant reduction in corticospinal excitability with baclofen compared to placebo (Drug: *F*1,17 = 4.929 *P* = 0.040). There was also a significant effect of Time (*F*_2,34_ = 12.3, *P* < 0.001) driven by a dip in excitability at 2 h PA, although no significant Drug by Time interaction (*F*_2,34_ = 0.6271, *P* = 0.540) (Fig. 4A).

A paired samples *t* test performed on the baselined measures at 6 h PA showed that the baclofen-induced difference in corticospinal excitability was no longer significant after drug washout (*t*_17_ = 0.988, *P* = 0.337).

#### No effect of baclofen on TMS-assessed synaptic GABA_A_ or GABA_B_ mediated inhibition

We then went on to investigate the effects of baclofen on our TMS measures of GABA? and GABA_A_ activity. The normalized measures from the three timepoints during the AM session were analysed using a repeated measures ANOVA with a within-subjects factor of Drug (baclofen, placebo) and a within-subjects factor of Time (1 h PA, 2 h PA and 3 h PA). Neither SICI_2.5 ms_, nor LICI_150 ms_ were significantly affected by baclofen during the AM session (SICI_2.5 ms_: *F*_1,17_ = 0.263, *P* = 0.615; LICI_150ms_: *F*_1,14_ = 0.245, *P* = 0.628). There were also no significant changes in these measures with Time through the session (SICI_2.5 ms_: *F*2,34 = 0.550, *P* = 0.582; LICI_150 ms_: *F*2,28 = 2.29, *P* = 0.120), nor any interaction between Drug and Time (SICI_2.5 ms_: *F*_2,34_ = 1.612, *P* = 0.214; LICI_150 ms_: *F*_2,28_ = 0.968, *P* = 0.392) (Fig. 4B and C).

Finally, paired samples *t* tests revealed that there were no differences between the drug conditions at the 6 h PA timepoint (SICI_2_._5ms_: *t_17_* = 0.2495, *P* = 0.192; LICI_150ms_: *t_14_* = 0. 557, *P* = 0.587).

### Relationship between baclofen-induced GABA_B_ change and retention parameter

Finally, given the significant effects of baclofen on visuomotor learning, as well as the known effects of baclofen on GABA_B_ activity, we performed an exploratory analysis to investigate whether the effect of baclofen on behaviour could be explained by the drug-induced degree of change in GABA_B_ activity on a subject-by-subject basis.

We demonstrated a negative relationship between placebo-corrected LICI_150 ms_, during peak plasma concentration, and placebo-corrected retention parameter from the state space model (*r*10 = 0.782, *P* = 0.004). No other correlations with placebo-corrected LICI_150 ms_ reached significance (VML AM FB-on: *r*_10_ = 0.081, *P* = 0.813; VML AM FB-off: *r*_10_ = -0.409, *P* = 0.212; VML learning parameter, *r*_10_ = –0.343, *P* = 0.302) (Fig. 4D). Again, the significant correlation met the Bonferroni corrected significance level (α = 0.013). The correlation between the placebo-corrected retention parameter and LICI_150 ms_ also remained if the effect of Day was removed from the former (*r*_10_ = 0.684, *P* = 0.005) (i.e. by calculating the residuals of the retention parameters from a model containing only the effect of day, and then taking the difference of placebo and baclofen residuals).

### Baclofen did not impair memory or affect mood, and the study was well-blinded

There were no baseline differences in either the alertness mood component (paired samples *t* test: *t*_19_ = 1.483, *P* = 0.154) or the hedonic mood component (*t*_19_ = 0.394, *P* = 0.698), and so the mood measures from the subsequent timepoints were corrected for baseline. There were no significant differences in the changes in either alertness (paired samples *t* test: *t*_19_ = 1.700, *P* = 0.105) or hedonic mood (*t*_19_ = 0.507, *P* = 0.618) at the 1 h PA timepoint between the drug conditions.

Baclofen did not affect participant scores on either of the AVLT memory measures [LMM analysis, fixed effects of Drug (Baclofen, placebo), Day (1, 2), random effect of Participant] (AVLT immediate recall: *F*_1,16.3_ = 1.894, *P* = 0.187; AVLT delayed/immediate ratio: *F*_1,16.8_ = 0.016, *P* = 0.899). There were also no significant effects of Day (AVLT immediate: *F*_1,16.3_ = 0.035, *P* = 0.854; AVLT delayed/immediate ratio: *F*_1,16.8_ = 1.912, *P* = 0.185).

Similarly, we found no effect of Drug or Day on participant’s forward digit span (Drug: *F*_1,18_ = 0.000, *P* = 1.000; Day: *F*_1,18_ = 0.095, *P* = 0.761). However, the same analysis revealed a significant effect of Drug on backwards digit span (*F*_1,18_ = 8.691, *P* = 0.009) driven by an increase in the number of correctly recalled digits in the Baclofen condition. There was no significant effect of Day (*F*_1,18_ = 1.893, *P* = 0.186).

The study was well-blinded for both the participants and experimenters [Participants: baclofen BI = -0.3 (confidence interval = -0.78 to +0.18), placebo BI = 0.3 (confidence interval = -0.18 to +0.78); experimenters: baclofen BI = -0.1 (confidence interval = -0.53 to +0.33), placebo BI = -0.2 (confidence interval = -0.57 to +0.17). Although the participants scores are <±0.2, the negative value for baclofen and positive value for placebo indicates that participants had an overall tendency to believe they took placebo on both days.

## Discussion

The present study aimed to identify whether a single dose of the GABA_B_ agonist baclofen, equivalent to that normally taken by patients multiple times a day, impacted motor learning in healthy humans. We found that baclofen caused significant impairments to the learnt visuomotor aftereffect, and also reduced retention of the learnt mapping. To identify the contribution of different processes underlying the observed behaviour on the VML task, a state-space model was applied to the data producing learning rate and retention parameters. The baclofen-induced change in the state-space model retention parameter was correlated with the degree that baclofen increased TMS-measured GABA_B_ mediated inhibition. Baclofen administration did not cause significant changes to sequence learning ability as measured using the SRTT, although baclofen-induced reductions in visuomotor aftereffect correlated with a baclofen-induced impairment of learning of the SRTT.

### Baclofen caused reductions in visuomotor aftereffects and retention

Participants performed the visuomotor learning task to assess learning and retention of a transformation in visuomotor mapping. Across conditions, participants showed significant learning of the transformation required to counteract a perturbation, as indicated by reducing the angular error across the AM FB-on blocks and increasing the aftereffect expression (increasing angular error) across the AM FB-off blocks. Across the AM session, the aftereffect remained labile, tending to decrease towards the end of each block (Fig. 2A). This is in contrast to a similarly designed study of prism adaptation learning, where the aftereffect stabilized after repeated prism exposure (O’Shea *et al*. 2017). This difference may be a result of the more immersive nature of prism adaptation, where the visuomotor transformation occurs across the whole visual field compared to only on-screen in our VML paradigm, potentially allowing for increased crystallization of the novel visuomotor mapping.

Performing the PM session of the VML task resulted in significant washout of the learnt mapping across the groups (error reduction in the FB-on blocks), in a pattern similar to that previously observed in prism adaptation washout (O’Shea *et al*. 2017). There was a significant reduction in the degree of expression of visuomotor aftereffect with baclofen, as measured by a smaller error in the AM FB-off blocks. Baclofen also resulted in an increased loss of the learnt visuomotor mapping during unlearning, as indicated by lower error in the PM FB-on blocks.

Visual examination of the PM session data (Fig. 2D) shows that the baclofen and placebo curves appear to follow a similar initial pattern of washout, although this diverges after a few blocks. A speculative suggestion for this pattern may be the differential effect of baclofen on visuomotor learning and retention processes. The behaviour observed in the initial blocks of the task could be reflective of learning the no-perturbation ‘normal’ mapping, a process not influenced by baclofen, whereas behaviour in the later stages of the task may be driven by an expression of any residual perturbed mapping retention, which could be reduced with baclofen.

### Increasing GABA_B_ mediated inhibition leads to a decrease in retention of the learnt transformation in visuomotor mapping

This result was further probed by applying a state-space modelling approach to produce a learning rate and retention parameter for each participant on each day of testing. The baclofen-induced change in the retention parameter correlated with the degree of baclofen-induced change in GABA_B_ receptor activation, where greater increases in GABA_B_ activity were related to decreases in the retention parameter. It has previously been reported that a greater MRS-measured excitation: inhibition ratio within M1 (i.e. less inhibition) is related to a greater weighing of retention processes in a prism adaptation paradigm (Petitet *et al*. 2018). Here, we build on this correlational link, to provide causal evidence that increasing GABAergic inhibition impairs retention of visuomotor learning.

Visuomotor learning and adaptation processes are considered to involve a network of brain regions including M1 and the cerebellum. The cerebellum’s highly organized structure (where Purkinje cells receive weak inputs from parallel fibres, perhaps carrying information about the body state, and strong inputs from climbing fibres, potentially indicating when unexpected errors occur) makes it well suited to learning through forward model computation. It has previously been suggested that, although the cerebellum is responsible for learning the transformation in visuomotor mapping, M1 may be more involved with retention (Galea *et al*. 2011). Although we recorded TMS measures of GABA_B_ activity within M1, we did not measure any neurophysiological changes within the cerebellum, where there are many baclofen sensitive GABA_B_ receptors (Wilkin *et al*. 1981). A change in activation at these receptors probably also influences the behavioural effects of baclofen, although further research is needed on this.

### No significant effect of baclofen on SRTT learning or retention

Participants performed the serial reaction time task as an assessment of motor sequence learning, which decreases MRS-quantified GABA within M1 (Chen *et al*. 2017; Floyer-Lea *et al*. 2006; Kolasinski *et al*. 2018). Across the conditions, participants demonstrated learning on the task, although baclofen did not appear to modulate learning or retention of the skill. There was, however, a significant correlation between the (non-significant) effect of baclofen on SRTT learning and the effect on visuomotor aftereffect, potentially suggesting that the effect of baclofen on these processes may be related, although our measure of sequence learning may be a less sensitive measure than visuomotor learning. Therefore, although the present study provides no significant evidence of an impairment in sequence learning with baclofen, this does not necessarily mean that it will not interfere with functional recovery after stroke, which is probably underpinned by a complex interaction of learning and adaptation mechanisms (Krakauer, 2015).

### Baclofen did not impair memory or alter mood

Learning on both the SRTT and the VML task involves a mixture of implicit and explicit processes (Robertson, 2007; Sülzenbrück & Heuer, 2009). It could therefore be argued that a baclofen-induced effect on memory or alertness could influence the explicit aspects of learning on these tasks, changing performance without affecting any motor learning processes. To mitigate this argument, we assessed participants memory on two different working memory tasks, the AVLT and Digit Span tasks, and recorded participants mood at multiple timepoints. Baclofen did not alter participant’s hedonic mood or alertness, nor did it alter performance on the AVLT or on the forward digit span. We did, however, find baclofen-induced improvement on the backward digit span score. This highly unexpected result requires follow-up investigation, and any attempts to explain it here would be highly speculative. However, overall, these results suggest that the dose of baclofen used here was not detrimental to participants alertness and memory.

Therefore, these factors probably do not explain the impairment in retention on the VML task.

### Baclofen resulted in group-level decreases in corticospinal excitability

Baclofen significantly decreased corticospinal excitability during peak-plasma concentration, which returned to baseline after drug washout. We found no overall group-level effects on TMS-measured GABA_A_ or GABA_B_ receptor activation.

Previous literature has reliably reported baclofen- induced increases in GABA_B_ mediated inhibition (McDonnell *et al*. 2006, 2007; Müller-Dahlhaus *et al*. 2008), with some papers also showing a decrease in GABA_A_ mediated inhibition (McDonnell *et al*. 2006; Ziemann *et al*. 1996). These studies used 50 mg of baclofen, and so the lower dose used in this case may be the reason for the lack of overall group effect on LIC1_150 ms_ measured GABA_B_ inhibition.

Changes to corticospinal excitability as found in the present study have not generally been demonstrated elsewhere in the literature (McDonnell *et al*. 2006, 2007); however, baclofen has been shown to decrease the corticospinal excitability changes induced by paired-associative stimulation (McDonnell *et al*. 2007). The 2 h PA and 3 h PA timepoints immediately followed performance of motor tasks, potentially meaning that baclofen impacts task performance effects on corticospinal excitability, rather than influencing resting corticospinal excitability *perse*. Again, differences in dosage could also play a role; a higher dose may counterintuitively have a lesser effect on corticospinal excitability if it were to cause compensatory or homeostatic processes to become active.

### Clinical relevance

The most common clinical use of baclofen is as a treatment for spasticity, where it acts as a muscle relaxant via agonism of GABA_B_ receptors in the spinal cord (Davidoff, 1985). However, it is well established that baclofen also acts on GABA_B_ receptors in the brain (Deguchi *et al*. 1995; Hill and Bowery, 1981; McDonnell *et al*. 2006). Here, we found that a single low dose of baclofen, equivalent to the dose taken several times a day by most patients, can disrupt motor learning in healthy individuals. If baclofen were to similarly impact similar processes involved in motor rehabilitation, this could have deleterious consequences for functional recovery in stroke and brain injury patients.

There are several reasons why the conclusions from the present study cannot be directly extended to patients, most of whom take baclofen chronically for years at a time, and where lesions or degeneration cause changes to brain physiology. Furthermore, the processes involved in sequence or visuomotor learning, used in the present study as model motor learning processes, are probably not directly involved in motor rehabilitation (Krakauer & Carmichael, 2017). However, performing pharmacological interventional studies in patients, when the drug is hypothesized to have negative impacts on outcomes, is ethically difficult, and retrospective or observational studies face issues with heterogeneity and comorbities in the patient populations. The evidence presented here may be used to motivate future retrospective or observational studies and, taken together, could provide insights into whether baclofen administration influences motor rehabilitation, as well as whether it should be prescribed with the same caution as other GABA agonists (Hesse & Werner, 2003)

### Limitations

The present study employed a within-subjects design to examine the effect of baclofen on two different behavioural tasks and TMS-measured physiology. This choice of design was made to minimize variability in the measures, all of which have an inherent high degree of inter-subject variability, across the testing days. Repeating behavioural tasks in the same participants introduce the potential for savings (i.e. improved or accelerated re-learning on second exposure to a task). However, efforts were made to minimize the effect of testing day, by counterbalancing the order of the drug administration, leaving 1 week between testing sessions, and by including Day as an effect in behavioural analyses.

## Conclusions

A single low dose of baclofen resulted in reduced visuomotor aftereffect and increased washout (i.e. less retention, of a learnt transformation in visuomotor mapping). State-space modelling of the behavioural data produced retention and learning parameters, and this retention parameter correlated with baclofen-induced increases in GABA_B_ inhibition, where a greater increase in inhibition was related to reduced retention. This result confirms the importance of GABAergic inhibition in this form of visuomotor learning and may have implications for functional rehabilitation in patients who are taking baclofen.

## Supplementary Material

Supplemental

## Data Availability

The data that support the findings of this study are available from the corresponding author upon reasonable request.

## Key points

- Baclofen is a GABA_B_ agonist prescribed as a treatment for spasticity in stroke, brain injury and multiple sclerosis patients, who are often undergoing concurrent motor rehabilitation.
- Decreasing GABAergic inhibition is a key feature of motor learning and so there is a possibility that GABA agonist drugs, such as baclofen, could impair these processes, potentially impacting rehabilitation.
- Here, we examined the effectof 10 mg of baclofen, in 20 young healthy individuals, and found that the drug impaired retention of visuomotor learning with no significant effect on motor sequence learning.
- Overall baclofen did not alter transcranial magnetic stimulation-measured GABA_B_ inhibition, although the change in GABA_B_ inhibition correlated with aspects of visuomotor learning retention.
- Further work is needed to investigate whether taking baclofen impacts motor rehabilitation in patients.
